# Time trends in income-related disparity in incidence of acute coronary syndrome

**DOI:** 10.1093/eurpub/ckad139

**Published:** 2023-08-07

**Authors:** Amalie H Simoni, Kriatian H Kragholm, Henrik Bøggild, Svend E Jensen, Jan B Valentin, Søren P Johnsen

**Affiliations:** Department of Clinical Medicine, Danish Center for Health Services Research (DACS), Aalborg University, Denmark; Unit of Clinical Biostatistics, Aalborg University Hospital, Denmark; Department of Cardiology, North Denmark Regional and Aalborg University Hospital, Denmark; Unit of Clinical Biostatistics, Aalborg University Hospital, Denmark; Public Health and Epidemiology Group, Department of Health Science and Technology, Aalborg University, Denmark; Department of Cardiology, Aalborg University Hospital, Denmark; Department of Clinical Medicine, Aalborg University, Denmark; Department of Clinical Medicine, Danish Center for Health Services Research (DACS), Aalborg University, Denmark; Department of Clinical Medicine, Danish Center for Health Services Research (DACS), Aalborg University, Denmark

## Abstract

**Background:**

Higher incidence of acute coronary syndrome (ACS), among those with lower income, has been recognized in the most recent decades. Still, there is a paucity of data on temporal changes. This study aims to investigate 20-year time trends in income-related disparity in the incidence of ACS in Denmark.

**Methods:**

This Population‐based repeated cross-sectional study included all patients with first-time ACS, aged ≥20 years, registered in the Danish National Patient Registry 1998–2017. Aggregated sociodemographic data for the Danish population was accessed from Statistics Denmark. Yearly incidence rates (IR) and incidence rate ratios (IRR), with the highest-income quartile as a reference, were standardized using cell-specific personal equivalent income according to year, sex and age group with 95% confidence intervals. Interaction analysis was executed for differences in IR of ACS between the lowest- and highest-income quartile over time.

**Results:**

A total of 220 070 patients hospitalized with ACS from 1998 to 2017 were identified. The yearly standardized ACS IRs decreased in all income quartiles. However, the IR remained higher in the lowest-income quartile compared to the highest for both men [1998: IRR 1.45 (95% confidence interval, CI 1.39–1.52) and 2017: 1.47 (1.40–1.54)] and women [1998: IRR 1.73 (1.64–1.82) and 2017: 1.76 (1.65–1.88)]. Interaction analysis showed that over the period the difference in IR between the lower- and the highest-income quartile decreased with 1–5 ACS cases per 100 000 person-year.

**Conclusion:**

Income-related disparity in the incidence of ACS was present in Denmark between 1998 and 2017. Despite a marked overall decrease in the yearly ACS incidence, the extent of income-related disparity remained unchanged.

## Introduction

Acute coronary syndrome (ACS) is the acute presentation of atherosclerosis, including myocardial infarction (MI) and unstable angina pectoris (UAP) and presents a major public health issue, ranking as the leading cause of morbidity and mortality globally.^1^^,^^2^ Mainly across high-income countries, the epidemiology of ACS has changed during the last century, by targeting the traditional cardiovascular risk factors in prevention.^3–5^ For almost 35 years, it has been acknowledged that a lower socioeconomic position (SEP), represented by income, education, and occupation, is generally associated with a higher incidence of ACS.^6^^,^^7^ This health disparity implies an economic burden since large groups of subjects may have poor health to contribute to societal production, and furthermore, it counteracts the individual’s ability to live a free life.^8^ All indicators of SEP represent a comprehensive context, acknowledging different pathways for disparity in health (e.g. resources, knowledge or network) with changing importance during the life course.^9^ Personal equivalent income is the indicator that most exactly measures the availability of personal material resources and is considered to be a robust indicator throughout professional life and retirement.^9^^,^^10^

The total age-adjusted incidence of ACS has decreased across high-income countries since the 1980es.^3^^,^^5^ However, considerable disparity in ACS incidence, according to various measures of SEP in favour of individuals with a higher SEP has been reported in most studies investigating this matter in the last decades.^11^^,^^12^ Studies have investigated time trends in socioeconomic disparity in the incidence of hospitalized MI and out-of-hospital fatal MI, according to education, occupation and composite measures, without identifying improvements.^13–18^ Similarly, no significant change was identified in socioeconomic disparity in the incidence of UAP.^18^ One study by Geyer et al. evaluated 10-year trends in personal income-related disparity in the incidence of hospitalized MI, using German regulatory data on 2.5 million adults. They found a narrowed relative income-related disparity for MI in men, but for women, the pattern was unclear.^19^ However, updated studies including high-quality validated nationwide data on hospital admissions for ACS and out-of-hospital fatal ACS could reveal that the disparity could be underestimated due to possible barriers in hospital admissions. Thus, actual changes in trends of socioeconomic disparity may have been concealed.

The World Health Organization and the European Society of Cardiology recently emphasize that addressing people at high risk of ACS, based on SEP, needs improvement to strengthen prevention and reduce the need for hospitalization and interventions.^20^^,^^21^ Thus, updated evidence on temporal trends in income-related disparity in ACS is warranted. Although the Danish health service system provides tax-supported healthcare for the entire population, health equity is a health policy priority and the country has been leading in improving ACS care,^22^ it has not yet been documented whether the income-related disparity in ACS incidence has changed over the last decades.^23^

The present study aimed to investigate time trends in income-related disparity in the incidence of hospitalized ACS and out-of-hospital fatal ACS, in Denmark from 1998 to 2017.

## Methods

### Study design and settings

This population-based repeated cross-sectional study was conducted using data from national administrative registries, covering all contacts to the healthcare sector and information on SEP, on individual or aggregated levels.^10^^,^^23–25^ The study was reported according to the guidelines for The Reporting of studies Conducted using Observational Routinely-collected health Data (RECORD), which is an extended version of the STrengthening the Reporting of OBservational studies in Epidemiology (STROBE) statement (Supplementary appendix S1)^26^^,^^27^ and registered at the institutional review board (Journal number: 2019-899/10-0429).

### Data sources

Patient-based data from several Danish nationwide registries were accessed through the Danish Health Data Authorities and Statistics Denmark. Statistics Denmark is a state institution, hosting multiple administrative registers from governmental agencies, including healthcare information, demography and personal income on individual or aggregated levels.^10^^,^^24^ The Danish National Patient Registry (DNPR) holds data on diagnoses for all inpatients discharged from Danish non-psychiatric hospitals since the database was established in 1977. Additionally, DNPR contains information from emergency departments and outpatient specialty clinics since 1995.^23^ For each patient contact, one primary and optional secondary diagnoses are recorded, classified according to the *International Classification of Diseases* 8th revision (ICD-8), since 1977, and 10th revision (ICD-10), since 1994. The Danish Register of Causes of Death (DRCD) holds information on the causes of death according to ICD-10 and the status of collection of cause of death certificates.^28^ The Danish Civil Registration System is an administrative register established in 1968, which contains individual-level information on all Danish residents, including age, sex and vital status, allowing register linkage and follow-up.^25^

### Source population

The source population was defined as the entire Danish population aged ≥20 years from 1998 to 2017, with available aggregated sociodemographic data in the STATBANK at Statistics Denmark, including sex, age and yearly personal equivalent income.^10^^,^^24^ Hence, the coronavirus 2019 (COVID-19) pandemic has no impact on the ACS hospitalizations in the present study.^29^

### Outcome

The study outcome was incident ACS. All patients aged ≥20 years with an ACS diagnosis from 1998 to 2017 were identified from nationwide population-based data on hospital admissions in the DNPR according to ICD-10 (MI: I21* and UAP: I200*), based on admission date and linked to sociodemographic data on an individual level.

Patients without an address in Denmark the year before and the year of the ACS diagnosis, without available income data, or with a negative taxable personal equivalent income were excluded to align the patient population with the background population available in the STATBANK data. Additionally, patients were excluded if they had any previous ACS diagnosis according to ICD-10 (MI: I21* and UAP: I200*) or ICD-8 (MI: 410* or 411* and UAP: 413*) since 1977, to ensure that only incident ACS events were included. Furthermore, individuals with out-of-hospital fatal ACS (I21* or I200*) and sudden death with unknown or unspecified cause (R999, R961, R989 or R990) in the DRCD, were included in a supplementary analysis if they had no previous ACS diagnosis.

### Exposure

The exposure in the present study was SEP based on yearly personal equivalent income. Yearly personal equivalent income statistics for the entire Danish population were available from Statistics Denmark in nominal prices, collected from the Danish tax authorities’ administrative registers linked to the population register. The yearly equivalent income was available in aggregated income tables according to year, sex and age group (in 5-year intervals from 20 to 79 years and all above 80 years).^10^^,^^24^ Individual-level sociodemographic data representing the respective year before the ACS were identified from Statistics Denmark and individually linked to the ACS patients. Sociodemographic data included sex, age and equivalent income. The personal equivalent income was provided by Statistics Denmark to enable comparability across different household sizes.^10^ The measurement is based on a modified scale from the Organization for Economic Cooperation and Development and redistributes the income equally among all household members.^10^

### Statistics

Statistical analysis and graphs were performed in Stata v. 16.0 (StataCorp. 2019; Stata Statistical Software: Release 16. StataCorp LLC, College Station, TX, USA). Individual characteristics were presented for the primary ACS population as medians and interquartile ranges or frequencies and percentages where appropriate. This was presented for five 4-calendar-year intervals (1998–2001, 2002–05, 2006–09, 2010–13 and 2014–17), to identify whether the covariates for the ACS patients differed according to calendar time.

To identify time trends in income-related disparity in the incidence of ACS, direct standardized incidence rates (IR) were calculated using cell-specific equivalent income quartiles for the year before ACS. The applied income quartiles were standardized, according to income year, sex and age group.^24^ The yearly direct standardized IRs were presented as ACS cases pr. 100 000 person-years stratified by sex. Standardized incidence rate ratios (IRR), using individuals in the highest-income quartile as the reference, were computed and graphically illustrated stratified by sex. Confidence intervals (CI) at 95% were estimated using a formula presented by Julious et al.^30^ The standardized IRs were compared for change in differences over time between the lower- with the highest-income quartiles by interaction analysis using random-effects meta-regression to account for the standard errors of the aggregated data. Interaction coefficients were presented with 95% CIs.

### Supplementary analyses

Supplementary analyses were performed, stratifying the individuals into patients with MI or UAP diagnoses according to personal equivalized income quartiles. Additionally, supplementary analyses were performed on subjects with death registered as caused by ACS or with sudden death without any defined reasons, without any previous ACS-related hospital admissions, to ensure that possible changes in disparity in incidence over time were not caused by changes in disparity in procedures for hospital admissions or registrations.

## Results

**Figure 1:**
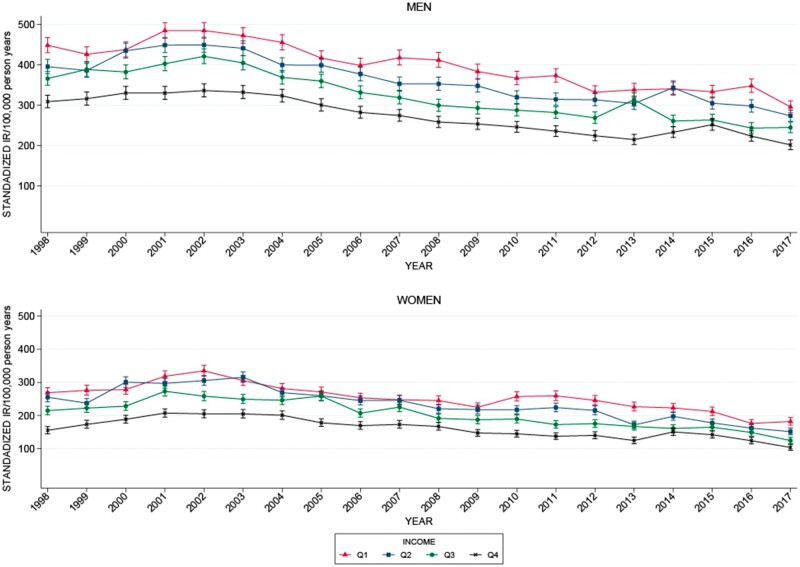
Standardized incidence rates of ACS pr. 100 000 person-years according to personal income quartile. The results are stratified by sex. Q: income quartile from low (1) to high (4), based on the income the year before the diagnosis. Analyses were standardized according to year, sex and age group

The Danish source population in 2016 included 4.4 million individuals aged ≥20 years. The median yearly personal equivalent income in 2016 was 233 300DKK (€31 365). Overall 1866 (0.8%) of the patients were excluded because they did not have an address in Denmark the year before and the year of the ACS diagnosis and 671 (0.3%) patients were excluded because equivalent income data was negative or missing. Thus, 220 070 patients aged ≥20, living in Denmark, with personal income data the year before diagnosis, were hospitalized with their first ACS diagnosis from 1998 to 2017 (table 1). The overall incidence of ACS decreased during the study period in all income quartiles (figure 1). The total yearly number of patients hospitalized with a first ACS was 9226 cases in 1998, peaked in 2002 at 13 920 cases, and decreased to 8427 in 2017.

**Table 1:** Characteristics of the patients hospitalized with acute coronary syndrome

	Calendar period					Total
	1998–2001	2002–05	2006–09	2010–13	2014–17	1998–2017
**ACS patients, *n***	49 735	52 177	42 838	39 026	36 294	220 070
**Age, median (IQR)**	70 (59–79)	70 (59–80)	69 (59–80)	69 (58–79)	69 (58–78)	70 (59–79)
**Male, *n* (%)**	30 572 (61%)	31 437 (60%)	26 219 (61%)	23 867 (61%)	22 927 (63%)	135 022 (61%)
**Diagnosis, *n* (%)**						
MI	40 731 (82%)	41 565 (80%)	34 474 (80%)	31 493 (81%)	30 053 (83%)	178 316 (81%)
UAP	9004 (18%)	10 612 (20%)	8364 (20%)	7533 (19%)	6241 (17%)	41 754 (19%)
**Income, n (%)**						
Q1	14 352 (29%)	15 080 (29%)	12 550 (29%)	11 861 (30%)	10 946 (30%)	64 789 (29%)
Q2	13 540 (27%)	14 077 (27%)	11 625 (27%)	10 597 (27%)	9815 (27%)	59 654 (27%)
Q3	12 023 (24%)	12 711 (24%)	10 104 (24%)	9079 (23%)	8403 (23%)	52 320 (24%)
Q4	9820 (20%)	10 309 (20%)	8559 (20%)	7489 (19%)	7130 (20%)	43 307 (20%)

ACS, acute coronary syndrome; IQR, interquartile ranges; MI, myocardial infarction; *n*, number of patients; *Q*, income quartile from low (1) to high (4), based on the income the year before the diagnosis; *UAP*, unstable angina pectoris.

### Incidence of ACS according to income

**Figure 2:**
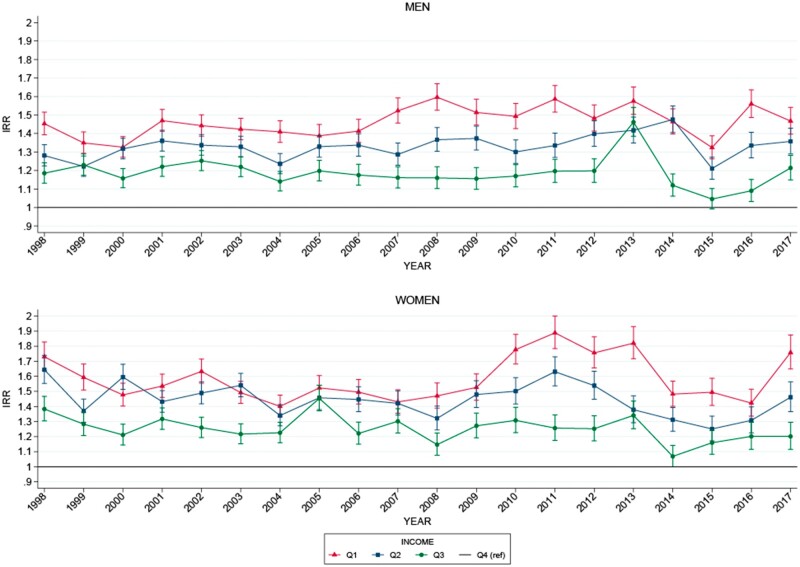
Standardized incidence rate ratio of ACS according to personal income quartile. The results are stratified by sex. Q: income quartile from low (1) to high (4), which is the reference group, based on the income the year before the diagnosis. Analyses were standardized according to year, sex and age group. Ref, reference group

Income-related disparity in the standardized IRs of ACS was present over the entire study period (figures 1 and 2 and table 2). The interaction analysis showed that the income-related disparity in the incidence of ACS decreased with 1–3 cases pr. 100 000 person pr. year from 1998 to 2017, when comparing any lower-income quartiles (Q1–3) to the highest-income quartile (Q4). This minor decrease in income-related disparity was statistically insignificant for all income groups, except for women in the second-lowest income quartile (Q2). The coefficients for interaction for men were Q1: −1.57, 95% CI (−4.06 to 0.92); Q2: −1.21, 95% CI (−3.69 to 1.28); Q3: −1.92, 95% CI (−4.41 to 0.56) compared to the highest-income quartile (Q4). Coefficients for interaction for women were Q1: −1.66, 95% CI (−4.06 to 0.74); Q2: −3.00, 95% CI (−5.38 to −0.60); Q3: −1.95, 95% CI (−4.33 to 0.44) compared to the highest-income quartile (Q4).

**Table 2:** Incidence rates and incidence rates ratios for ACS according to equivalent personal income quartile

	1998–2001	2002–05	2006–09	2010–13	2014–17	Total 1998–2017

Incidence rates/100 000 person-years						
Income	IR [CI]	IR [CI]	IR [CI]	IR [CI]	IR [CI]	IR [CI]
**Men**						
Q1	449 [440–459]	457 [448–467]	403 [394–412]	352 [344–361]	329 [321–336]	397 [393–401]
Q2	416 [407–425]	422 [413–431]	357 [349–366]	313 [305–320]	304 [296–311]	362 [358–365]
Q3	385 [376–394]	388 [380–397]	310 [303–318]	287 [279–294]	253 [246–259]	323 [320–327]
Q4	321 [313–329]	323 [315–331]	267 [260–274]	230 [223–236]	226 [220–233]	272 [269–276]
**Women**						
Q1	368 [362–374]	376 [370–382]	319 [314–325]	302 [296–307]	263 [258–268]	325 [322–327]
Q2	344 [338–350]	354 [348–360]	296 [290–301]	259 [254–264]	236 [231–240]	297 [295–299]
Q3	308 [303–314]	320 [315–326]	257 [252–262]	231 [226–236]	202 [197–206]	263 [261–265]
Q4	250 [245–255]	259 [255–265]	215 [211–220]	183 [179–187]	180 [176–184]	217 [215–219]

Incidence rate ratios						
Income	IRR [CI]	IRR [CI]	IRR [CI]	IRR [CI]	IRR [CI]	IRR [CI]
**Men**						
Q1	1.40 [1.37–1.43]	1.42 [1.39–1.45]	1.51 [1.48–1.54]	1.53 [1.50–1.57]	1.45 [1.42–1.49]	1.46 [1.44–1.47]
Q2	1.30 [1.27–1.32]	1.31 [1.28–1.34]	1.34 [1.31–1.37]	1.36 [1.33–1.39]	1.34 [1.31–1.38]	1.33 [1.31–1.34]
Q3	1.20 [1.17–1.23]	1.2 [1.18–1.23]	1.16 [1.13–1.19]	1.25 [1.22–1.28]	1.12 [1.09–1.15]	1.19 [1.17–1.20]
Q4	Ref	Ref	Ref	Ref	Ref	Ref
**Women**						
Q1	1.47 [1.45–1.50]	1.45 [1.43–1.47]	1.48 [1.46–1.51]	1.65 [1.62–1.68]	1.46 [1.43–1.49]	1.50 [1.48–1.51]
Q2	1.38 [1.36–1.40]	1.37 [1.34–1.39]	1.37 [1.35–1.40]	1.42 [1.39–1.44]	1.31 [1.29–1.34]	1.37 [1.36–1.38]
Q3	1.24 [1.21–1.26]	1.23 [1.21–1.26]	1.19 [1.17–1.22]	1.26 [1.24–1.29]	1.12 [1.10–1.15]	1.21 [1.20–1.22]
Q4	Ref	Ref	Ref	Ref	Ref	Ref

The results are stratified by sex. The IR and IRR are standardized according to year, sex and age group. ACS, acute coronary syndrome; *n*, number of patients; *Q*, quartile, IR, incidence rate; IRR, incidence rate ratio; *a*, adjusted; 95% CI, 95% confidence interval.

### Incidence of MI and UAP according to income

When stratifying the outcome according to MI and UAP diagnoses, income-related disparity was primarily found in MI incidence (Supplementary appendixes S2 and S3). The interaction analysis showed that the income-related disparity in the incidence of MI decreased with 1–3 cases pr. 100 000 person pr. year from 1998 to 2017, when comparing any of the lower-income quartiles (Q1–3) to the highest-income quartile (Q4). The income-related disparity in the incidence of UAP was less explicit, and no trends in change over time were identified from the interaction analysis (Supplementary appendixes S2 and S3).

### Incidence of hospitalized and fatal non-hospitalized ACS according to income

In addition to the hospitalized ACS patients, 30 947 fatal non-hospitalized ACS cases and 34 737 individuals with sudden death with an unknown or unspecified reason of death in the DRCD, without previously known ACS, were identified. When including the fatal non-hospitalized ACS cases and, also, when including individuals with sudden deaths for an unknown or unspecified reason, the time trends in income-related disparity were similar to the analysis restricted to the hospitalized ACS patients (Supplementary appendixes S4 and S5). Thus, income-related disparity in the incidence of ACS and deaths which could be related to the incidence of ACS was present across the entire study period. The disparity tended to slowly decrease, with a point estimate of 1–5 cases pr. 100 000 person pr. year, compared to the highest-income quartile (Q4) (Supplementary appendixes S4 and S5).

## Discussion

The present study found that income-related disparity in the incidence of ACS was present in Denmark from 1998 to 2017. Despite the large decrease in yearly incidence, the income-related disparity between the lowest and the highest-income quartiles only decreased with up to five cases pr. 100 000 person pr. year. These findings were robust across the supplementary analyses, except that it was not present in the UAP patients.

With the current speed, it will take many decades before the narrowing of maximum five ACS cases yearly pr. 100 000 persons will have any substantial clinical effect and the intention of eliminating the income-related disparity in the incidence of ACS is therefore not within reach.^20^^,^^31^ The disparity even temporarily increased for women from 2010 to 2013. Similar to the finding in the present study, Geyer et al. found income-related disparity in MI incidence between men with income below 40% and those with income above 80% of average national income, decreased by 4% pr. year from 2006 to 2015 in Germany.^19^ Other studies have applied alternative measures of socioeconomic disparity for the incidence of MI, including education, occupation and area-based composite measures of SEP.^13–18^ The socioeconomic disparity in the incidence of MI according to composite measures increased from 1990 to 2002 in Scotland and from 1999 to 2007 in the UK,^15^^,^^18^ and persisted from 1998 to 2007 in the Netherlands and from 2000 to 2012 in Australia.^14^^,^^17^ Studies using education as a proxy for SEP also found that the relative disparity did not change from 1987 to 2008 in Sweden, or 2001–2009 in Norway.^5^^,^^13^ Additionally, the socioeconomic disparity in MI incidence persisted from 1987 to 2010 in Sweden, measured by occupation status.^16^ Thus, it seems that socioeconomic disparity in the incidence of ACS is an unsolved issue across countries, regardless of the measure of SEP, with no explicit signs of clinical improvement in disparity during the last 30 years. This is problematic, in particular since a reduction of socioeconomic disparity in ACS incidence has the potential to improve disparity in overall mortality.^15^ However, it is an alleviating finding, probably related to better preventive strategies^32^^,^^33^ that the incidence of ACS decreased after 2002 within all four income quartiles in the present study, and thus, the disparity in incidence of ACS has not increased.

The present study found that income-related disparity in the incidence of hospitalized MI in Denmark from 1998 to 2017 was stronger than income-related disparity in the incidence of hospitalized UAP. MI and UAP present as acute coronary atherosclerosis, with ischaemic pain and severe dyspnoea symptoms, of diverse severity and both diagnoses require acute hospitalization and clinical investigations.^2^ Thus, ideally all MI and UAP diagnoses should be captured equally well in the DNPR. However, despite the severe and acute circumstances, possible different approaches to healthcare-seeking behaviour could imply fewer individuals with lower income seeking acute hospital care at symptom onset, especially for UAP.^34^ Opposite to the findings from the present study, Pearson-Stuttard et al. found socioeconomic disparity in the incidence of UAP in the UK from 1999 to 2007, without narrowing the disparity.^18^ Another explanation for the finding in the present study could be more favourable lifestyle factors and more effective primary prevention among individuals with a higher income.^15^^,^^17^ The proportion of daily smokers in the general population in Denmark decreased from 2000 to 2010, with a possible social gradient, declining more among persons with a higher SEP.^19^^,^^35^ This could imply atherosclerosis present as less severe acute events, resulting in a higher incidence of UAP but a lower incidence of MI among individuals with higher income. Randall et al., presented an increase in non-ST-elevated MI in Western Australia from 1993 to 2012 despite an overall decrease in the incidence of MI supposedly caused by improved primary prevention across the population, possibly improving the diagnosis for patients who could have had an ST-elevated MI.^17^

### Study strengths and limitations

The Danish national healthcare is tax-supported and 85% of all healthcare costs are free, including hospitalization and partial reimbursement of prescribed medication. Thus, income-related disparity in the incidence of ACS should not derive from economic barriers to access to health care. However, economic barriers could still exist, e.g. regarding the use of preventive prescribed medication. The DNPR is used as an administrative tool for healthcare planning, by monitoring the occurrence of disease and treatment, used for paying all private and public hospitals. Therefore, DNPR is considered complete regarding all hospital-based diagnoses in Denmark, which is a major strength of the present study.^23^ Although access to healthcare is theoretically equal across citizens in the Danish public-funded system, there might still be differences in diagnoses, due to approaches to healthcare-seeking behaviour and abilities to express symptoms and need for care.^16^^,^^23^ Thus, the DNPR may capture possible disparity in acute ACS admissions rather than the actual onset of population-based ACS incidence.^23^ To overcome this issue, fatal out-of-hospital ACS diagnoses were included in the DRCD, which contains all registered deaths that have occurred in Denmark. However, this registry is not used for healthcare planning and thus, is somewhat less precise. Therefore, individuals with a registered sudden unknown reason of death or no received attest in the DRCD were included in the supplementary analyses to investigate this limitation. To our knowledge, this approach has not previously been applied. The results were robust across the inclusion of ACS patients from DNPR and DRCD and deaths with an unknown cause, although the last analysis also involved an unknown number of individuals with other possible causes of death.^28^ This may be explained by the fact that the identified lack of clinically relevant reduction in income-related disparity in ACS incidence is a public health concern not isolated to disparity in hospital admissions. However, it is important to recognize that not all ACS are fatal despite the lack of acute hospital admission and care. Thus, some unrecognized MI, which may overall account for up to half of all MIs, are not captured in the DRCD or DNPR.^36^ However, if the income-related disparity is presented in the unrecognized ACS, the disparity in ACS incidence would be even more distinctive than presented in the present study.

Due to the structure of the available data, the information on covariates was rather limited. Hence, a possible mechanism of socioeconomic factors, such as education and occupation, could partially explain the disparity identified in the present study; however, the socioeconomic disparity is nonetheless distinctive. Additionally, no information was available on prescriptions for preventive drugs. The available covariates could not be person-linked but were standardized using cell-specific aggregated data, including data on yearly income quartile the year before the ACS diagnosis, sex and age group. The income data available in Statistics Denmark are collected as administrative data and have a large validity regarding taxable income.^10^^,^^24^ To reduce the impact of inflation over the long study period and take salary changes over calendar time into account, age and year-specific income equivalents, were applied in the adjusted analyses.^10^

## Conclusions

Income-related disparity in the incidence of ACS was present in Denmark every year from 1998 to 2017, both among men and women. The disparity was present both in hospitalized and fatal non-hospitalized ACS cases, but mainly for the MI diagnoses. Despite a large decrease in the yearly incidence of ACS, the income-related disparity in the incidence of ACS has remained virtually unchanged during the last 20 years. Thus, it seems that reducing socioeconomic disparity in the incidence of ACS is an unsolved issue in the Danish welfare state.

## Supplementary Material

ckad139_Supplementary_Data

## Data Availability

All data used in the present study were accessed through the Danish Health Data Authorities and Statistics Denmark.
