# Supplementary material for: Time trends in income-related disparity in incidence of acute coronary syndrome
Source: Eur J Public Health. 2023 Aug 7;33(5):778–84. doi: 10.1093/eurpub/ckad139 (PMC10567243; doi:10.1093/eurpub/ckad139)

European Journal of Public Health

SUPPLEMENTARY MATERIAL

Time Trends in Income-related Disparity in Incidence of Acute Coronary Syndrome

Amalie H. Simoni, Kristian H. Kragholm, Henrik Bøggild, Svend E. Jensen, Jan B. Valentin and Søren P. Johnsen.

THE RECORD STATEMENT – CHECKLIST

Appendix S1: The RECORD statement – checklist of items, extended from the STROBE statement, that should be reported in observational studies using routinely collected health data. ……………………………..………………2-5

SUPPLEMENTARY ANALYSES

Appendix S2: Time trends in income-related disparity in myocardial infarction…………………...……...…..……6-7

Appendix S3: Time trends in income-related disparity in unstable angina pectoris……………..……...…………...8-9

Appendix S4: Time trends in income-related disparity in hospitalised and fatal non-hospitalised ACS…..…....….10-11

Appendix S5: Time trends in income-related disparity in hospitalised and fatal non-hospitalised ACS and sudden deaths with an unknown or unspecified reason………………………………………………………………………12-13

THE RECORD STATEMENT – CHECKLIST

Appendix S1: The RECORD statement – checklist of items, extended from the STROBE statement, that should be reported in observational studies using routinely collected health data.

	No.	STROBE items	RECORD items	Location in manuscript where items are reported
**Title and abstract**				
	1	(a) Indicate the study's design with a commonly used term in the title or the abstract (b) Provide in the abstract an informative and balanced summary of what was done and what was found	RECORD 1.1: The type of data used should be specified in the title or abstract. When possible, the name of the databases used should be included.<br><br>RECORD 1.2: If applicable, the geographic region and timeframe within which the study took place should be reported in the title or abstract.<br><br>RECORD 1.3: If linkage between databases was conducted for the study, this should be clearly stated in the title or abstract.	Abstract
**Introduction**				
Background rationale	2	Explain the scientific background and rationale for the investigation being reported		Background
Objectives	3	State specific objectives, including any prespecified hypotheses		Aim
**Methods**				
Study Design	4	Present key elements of study design early in the paper		Study design and settings
Setting	5	Describe the setting, locations, and relevant dates, including periods of recruitment, exposure, follow-up, and data collection		Study design and settings,<br>Data sources,<br>Source population
Participants	6	*(a) Cohort study* **-** Give the eligibility criteria, and the sources and methods of selection of participants. Describe methods of follow-up<br>*Case-control study* **-** Give the eligibility criteria, and the sources and methods of case ascertainment and control	RECORD 6.1: The methods of study population selection (such as codes or algorithms used to identify subjects) should be listed in detail. If this is not possible, an explanation should be provided.	Source population,

		selection. Give the rationale for the choice of cases and controls<br>*Cross-sectional study* - Give the eligibility criteria, and the sources and methods of selection of participants<br><br>*(b) Cohort study* - For matched studies, give matching criteria and number of exposed and unexposed<br>*Case-control study* - For matched studies, give matching criteria and the number of controls per case	RECORD 6.2: Any validation studies of the codes or algorithms used to select the population should be referenced. If validation was conducted for this study and not published elsewhere, detailed methods and results should be provided.<br><br>RECORD 6.3: If the study involved linkage of databases, consider use of a flow diagram or other graphical display to demonstrate the data linkage process, including the number of individuals with linked data at each stage.	
Variables	7	Clearly define all outcomes, exposures, predictors, potential confounders, and effect modifiers. Give diagnostic criteria, if applicable.	RECORD 7.1: A complete list of codes and algorithms used to classify exposures, outcomes, confounders, and effect modifiers should be provided. If these cannot be reported, an explanation should be provided.	Exposure, Source population
Data sources/ measurement	8	For each variable of interest, give sources of data and details of methods of assessment (measurement).<br>Describe comparability of assessment methods if there is more than one group		Outcome,<br>Exposure
Bias	9	Describe any efforts to address potential sources of bias		Exposure,<br>Statistics
Study size	10	Explain how the study size was arrived at		Source population
Quantitative variables	11	Explain how quantitative variables were handled in the analyses. If applicable, describe which groupings were chosen, and why		Exposure,<br>Statistics
Statistical methods	12	(a) Describe all statistical methods, including those used to control for confounding<br>(b) Describe any methods used to examine subgroups and interactions<br>(c) Explain how missing data were addressed<br>(d) *Cohort study* - If applicable, explain how loss to follow-up was addressed<br>*Case-control study* - If applicable, explain how matching of cases and controls was addressed<br>*Cross-sectional study* - If applicable, describe analytical methods taking account of sampling strategy<br>(e) Describe any sensitivity analyses		Statistics,<br>Supplementary analyses

Data access and cleaning methods		..	RECORD 12.1: Authors should describe the extent to which the investigators had access to the database population used to create the study population.<br><br>RECORD 12.2: Authors should provide information on the data cleaning methods used in the study.	Data sources,<br>Source population,<br>Outcome,
Linkage		..	RECORD 12.3: State whether the study included person-level, institutional-level, or other data linkage across two or more databases. The methods of linkage and methods of linkage quality evaluation should be provided.	Source population,<br>Outcome,<br>Exposure
**Results**				
Participants	13	(a) Report the numbers of individuals at each stage of the study (*e.g.*, numbers potentially eligible, examined for eligibility, confirmed eligible, included in the study, completing follow-up, and analysed)<br>(b) Give reasons for non-participation at each stage.<br>(c) Consider use of a flow diagram	RECORD 13.1: Describe in detail the selection of the persons included in the study (*i.e.*, study population selection) including filtering based on data quality, data availability and linkage. The selection of included persons can be described in the text and/or by means of the study flow diagram.	Source population,<br>Results
Descriptive data	14	(a) Give characteristics of study participants (*e.g.*, demographic, clinical, social) and information on exposures and potential confounders<br>(b) Indicate the number of participants with missing data for each variable of interest<br>(c) *Cohort study* - summarise follow-up time (*e.g.*, average and total amount)		Table 1
Outcome data	15	*Cohort study* - Report numbers of outcome events or summary measures over time<br>*Case-control study* - Report numbers in each exposure category, or summary measures of exposure<br>*Cross-sectional study* - Report numbers of outcome events or summary measures		Table 1,<br>Incidence of acute coronary syndrome according to income
Main results	16	(a) Give unadjusted estimates and, if applicable, confounder-adjusted estimates and their precision (e.g., 95% confidence interval). Make clear which confounders were adjusted for and why they were included<br>(b) Report category boundaries when continuous variables were categorized		Table 2

		(c) If relevant, consider translating estimates of relative risk into absolute risk for a meaningful time period		
Other analyses	17	Report other analyses done—e.g., analyses of subgroups and interactions, and sensitivity analyses		Incidence of MI and UAP according to income, Incidence of hospitalized and fatal non-hospitalized ACS according to income.
**Discussion**				
Key results	18	Summarise key results with reference to study objectives		Discussion
Limitations	19	Discuss limitations of the study, taking into account sources of potential bias or imprecision. Discuss both direction and magnitude of any potential bias	RECORD 19.1: Discuss the implications of using data that were not created or collected to answer the specific research question(s). Include discussion of misclassification bias, unmeasured confounding, missing data, and changing eligibility over time, as they pertain to the study being reported.	Strengths and Limitations
Interpretation	20	Give a cautious overall interpretation of results considering objectives, limitations, multiplicity of analyses, results from similar studies, and other relevant evidence		Discussion
Generalisability	21	Discuss the generalisability (external validity) of the study results		Discussion
**Other Information**				
Funding	22	Give the source of funding and the role of the funders for the present study and, if applicable, for the original study on which the present article is based		Acknowledgements
Accessibility of protocol, raw data, and programming code	23	..	RECORD 22.1: Authors should provide information on how to access any supplemental information such as the study protocol, raw data, or programming code.	Contact corresponding author

Reference: [24] Benchimol EI, Smeeth L, Guttmann A, et al. The REporting of studies Conducted using Observational Routinely-collected health Data (RECORD) Statement. PLoS Med 2015; 12: 1–22.

SUPPLEMENTARY ANALYSES

Appendix S2: Time Trends in Income-related Disparity in Myocardial Infarction

Figure S2.1 Standardised Incidence rates of MI pr. 100.000 person-years according to personal income quartile

The results are stratified by sex. Abbreviations: MI: Myocardial Infarction, Q: Income Quartile from low (1) to high (4), based on the income the year before the diagnosis. Analyses were standardised according to year, sex and age-group.

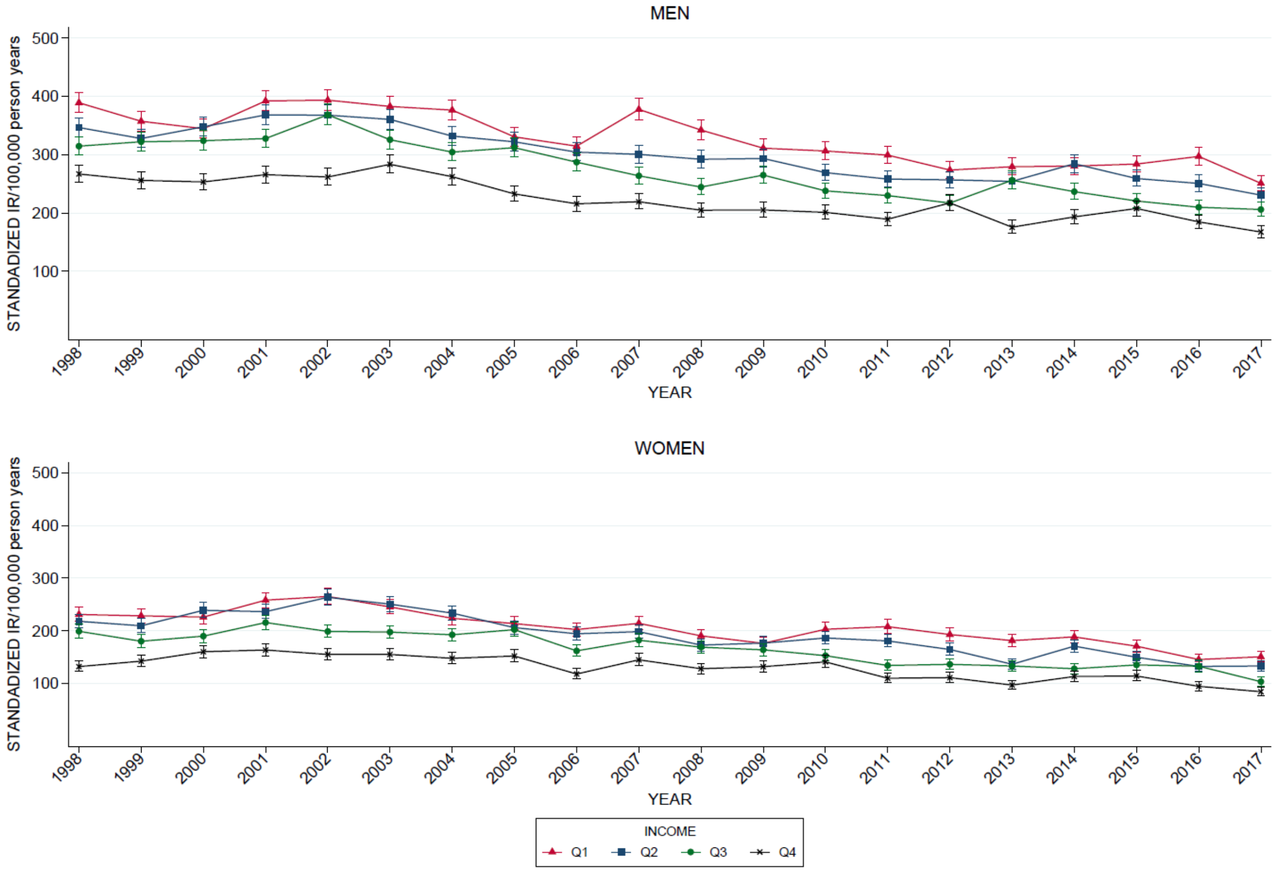

Table S2.1: Interaction analysis for time trends in incidence rates of MI according to personal income quartile.

	MEN	WOMEN
**Income**	**Coefficient [CI]**	**Coefficient [CI]**
Q1	-1.49 [-3.57; 0.59]	-1.46 [-3.16; 0.23]
Q2	-1.47 [-3.54; 0.61]	-2.58 [-4.27; -0.89]
Q3	-2.18 [-4.25; -0.11]	-1.61 [-3.29; 0.07]
Q4	*ref*	*ref*

The results are stratified by sex. Abbreviations: CI: 95% Confidence interval, MI: Myocardial Infarction, Q: quartile.

Figure S2.2 Standardised Incidence rate ratio of MI according to personal income quartile.

The results are stratified by sex. Abbreviations: MI: Myocardial Infarction, Q: Income Quartile from low (1) to high (4), which is the reference group, based on the income the year before the diagnosis, Ref: reference group. Analyses were standardised according to year, sex and age-group.

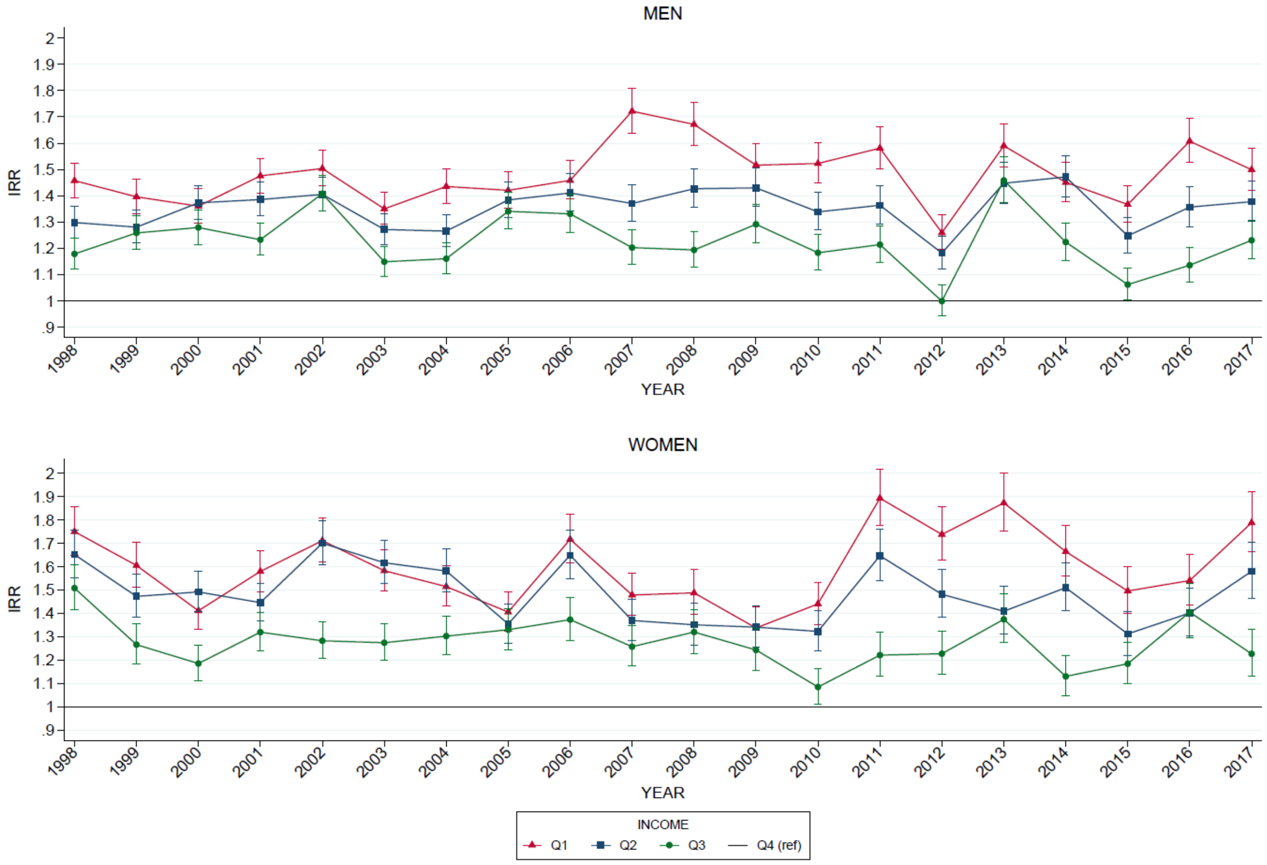

Appendix S3: Time Trends in Income-related Disparity in Unstable Angina Pectoris

Figure S3.1 Standardised Incidence rates of UAP pr. 100.000 person-years according to personal income quartile. *The results are stratified by sex. Abbreviations: UAP: Unstable Angina Pectoris, Q: Income Quartile from low (1) to high (4), based on the income the year before the diagnosis. Analyses were standardised according to year, sex and age-group.*

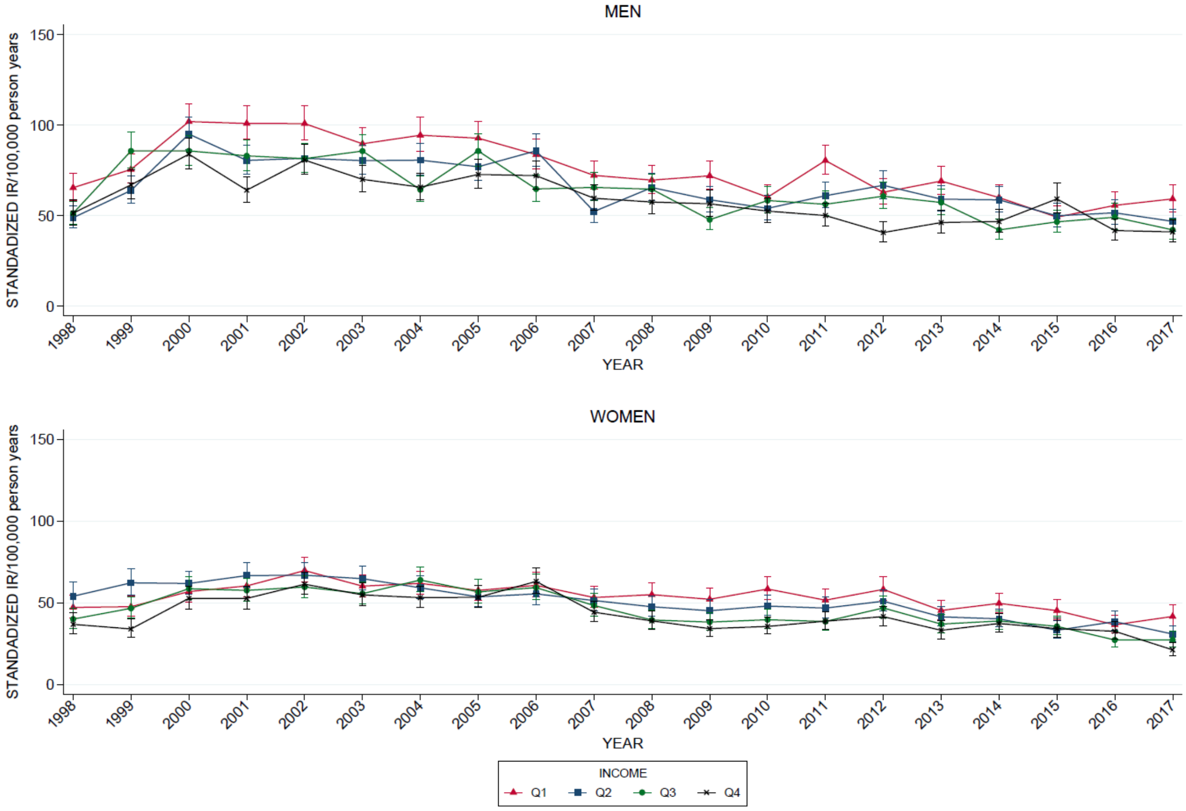

Table S3.1: Interaction analysis for time trends in incidence rates of UAP according to personal income quartile.

	MEN	WOMEN
**Income**	**Coefficient [CI]**	**Coefficient [CI]**
Q1	-0.39 [-1.57; 0.79]	0.39 [-0.40; 1.18]
Q2	0.20 [-0.97; 1.37]	-0.52 [-1.30; 0.28]
Q3	-0.37 [-1.54; 0.80]	-0.23 [-1.02; 0.55]
Q4	*ref*	*ref*

The results are stratified by sex. Abbreviations: CI: 95% Confidence interval, Q: quartile, UAP: Unstable Angina Pectoris.

Figure S3.2 Standardised Incidence rate ratio of UAP according to personal income quartile.

The results are stratified by sex. Abbreviations: UAP: Unstable Angina Pectoris, Q: Income Quartile from low (1) to high (4), which is the reference group, based on the income the year before the diagnosis, Ref: reference group. Analyses were standardised according to year, sex and age-group.

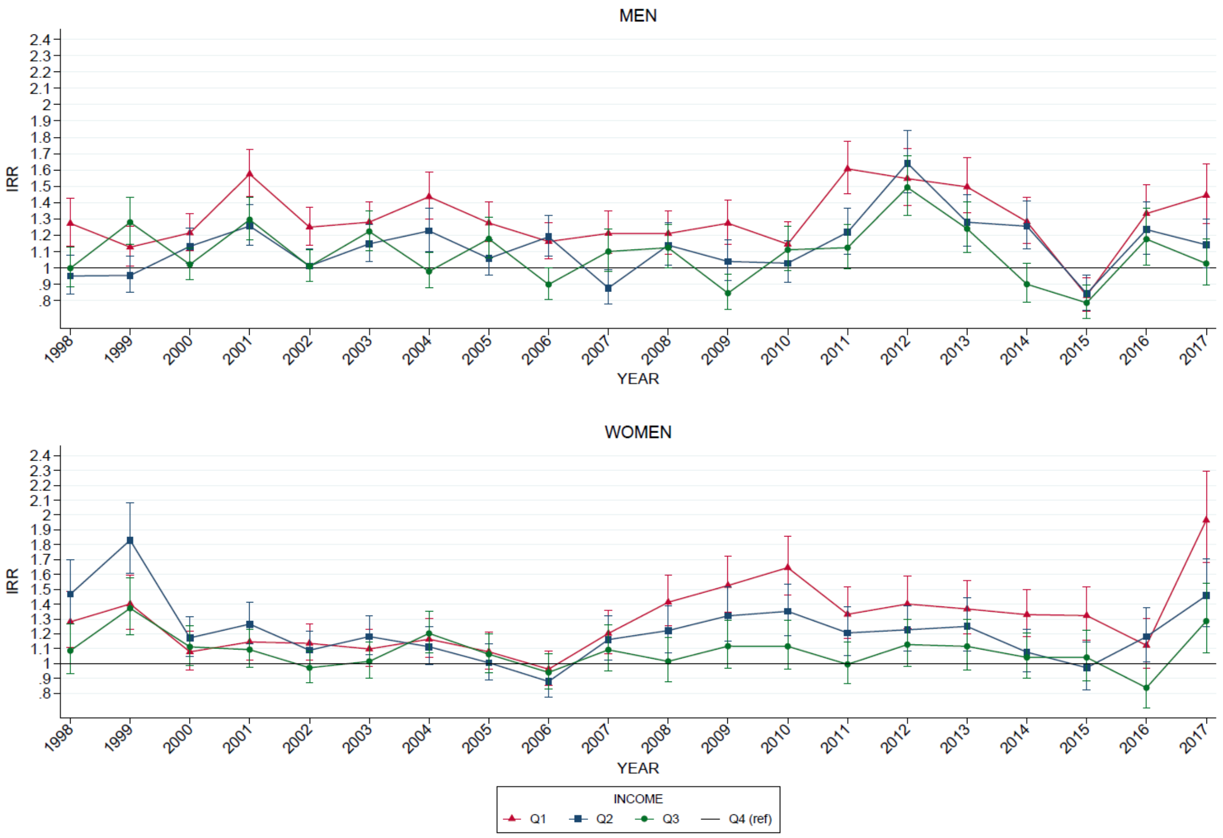

Appendix S4: Time Trends in Income-related Disparity in hospitalised and fatal non-hospitalised ACS

Figure S4.1 Standardised Incidence rates of hospitalised and fatal non-hospitalised ACS pr. 100.000 person-years according to personal income quartile. *The results are stratified by sex. Abbreviations: ACS: Acute Coronary Syndrome, Q: Income Quartile from low (1) to high (4), based on the income the year before the diagnosis. Analyses were standardised according to year, sex and age-group.*

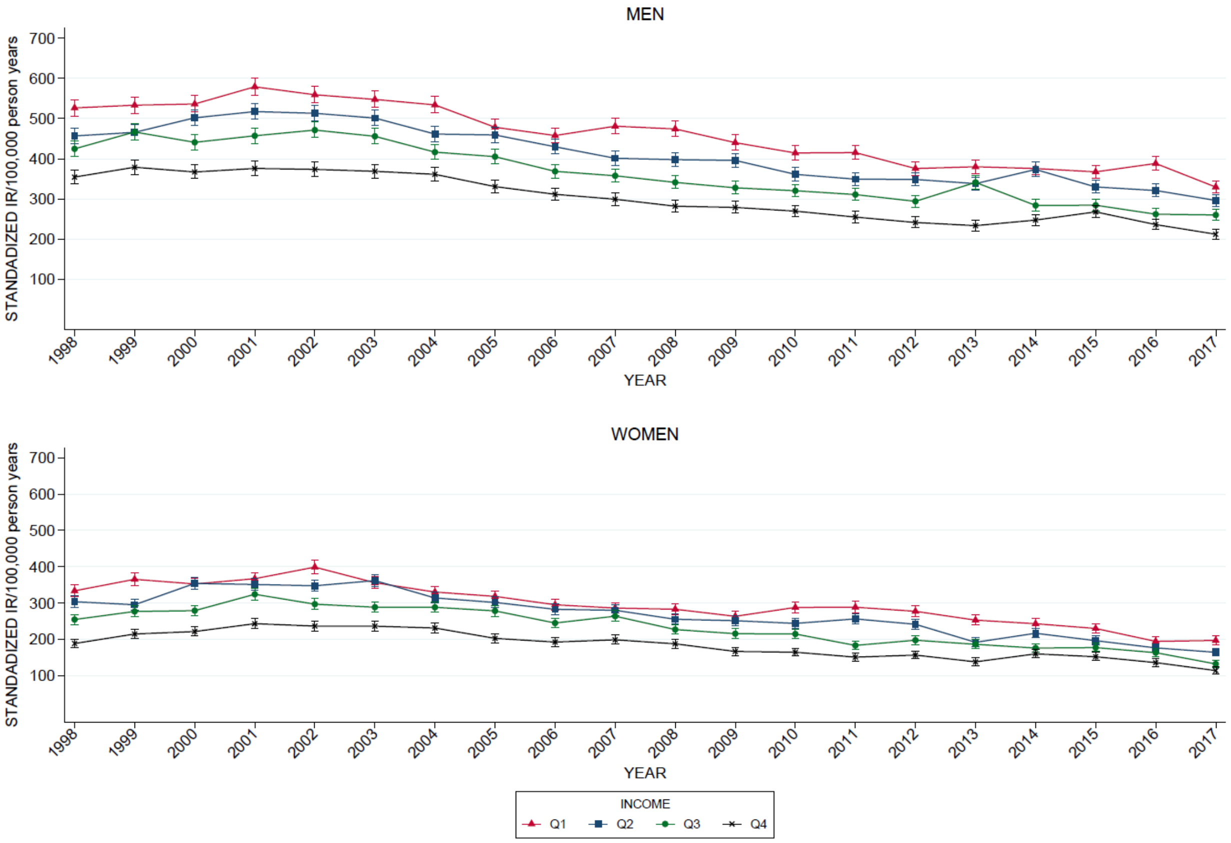

Table S4.1: Interaction analysis for time trends in incidence rates of hospitalised and fatal non-hospitalised ACS according to personal income quartile.

	**MEN**	**WOMEN**
**Income**	**Coefficient [CI]**	**Coefficient [CI]**
Q1	-3.00 [-5.59; -0.42]	-3.25 [-5.68; -0.81]
Q2	-1.88 [-4.46; 0.69]	-3.67 [-6.09; -1.24]
Q3	-2.44 [-5.01; 0.12]	-2.56 [-4.98; -0.14]
Q4	*ref*	*ref*

The results are stratified by sex. Abbreviations: ACS: Acute Coronary Syndrome, CI: 95% Confidence interval, Q: quartile,

Figure S4.2: Standardised Incidence rate ratio of hospitalised and fatal non-hospitalised ACS according to personal income quartile. *The results are stratified by sex. Abbreviations: ACS: Acute Coronary Syndrome, Q: Income Quartile from low (1) to high (4), which is the reference group, based on the income the year before the diagnosis, Ref: reference group. Analyses were standardised according to year, sex and age-group.*

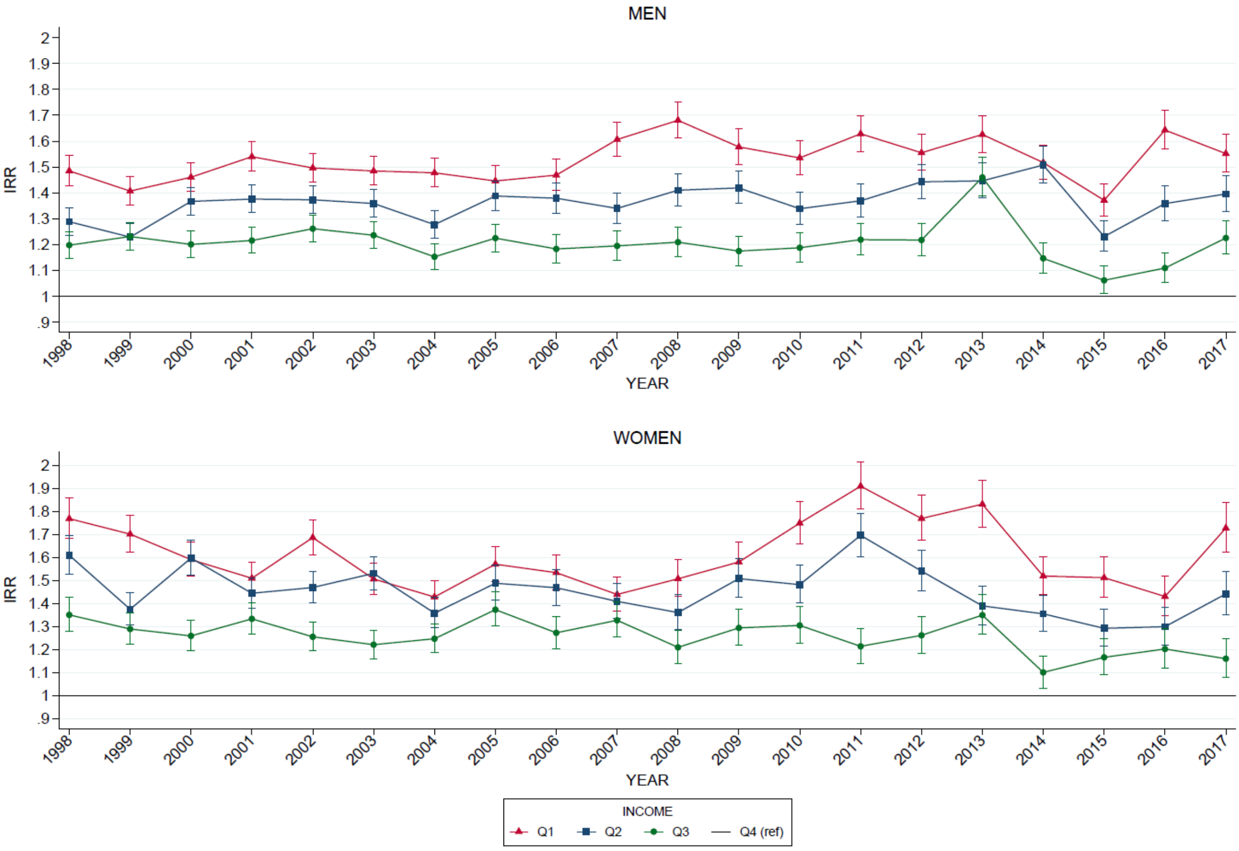

Appendix S5: Time Trends in Income-related Disparity in hospitalised and fatal non-hospitalised ACS and sudden deaths with an unknown or unspecified reason

Figure S5.1: Standardised Incidence rates of ACS and dead possibly related to ACS according to personal income quartile. *The results are stratified by sex. Abbreviations: ACS: Acute Coronary Syndrome, Q: Income Quartile from low (1) to high (4), based on the income the year before the diagnosis. Analyses were standardised according to year, sex and age-group.*

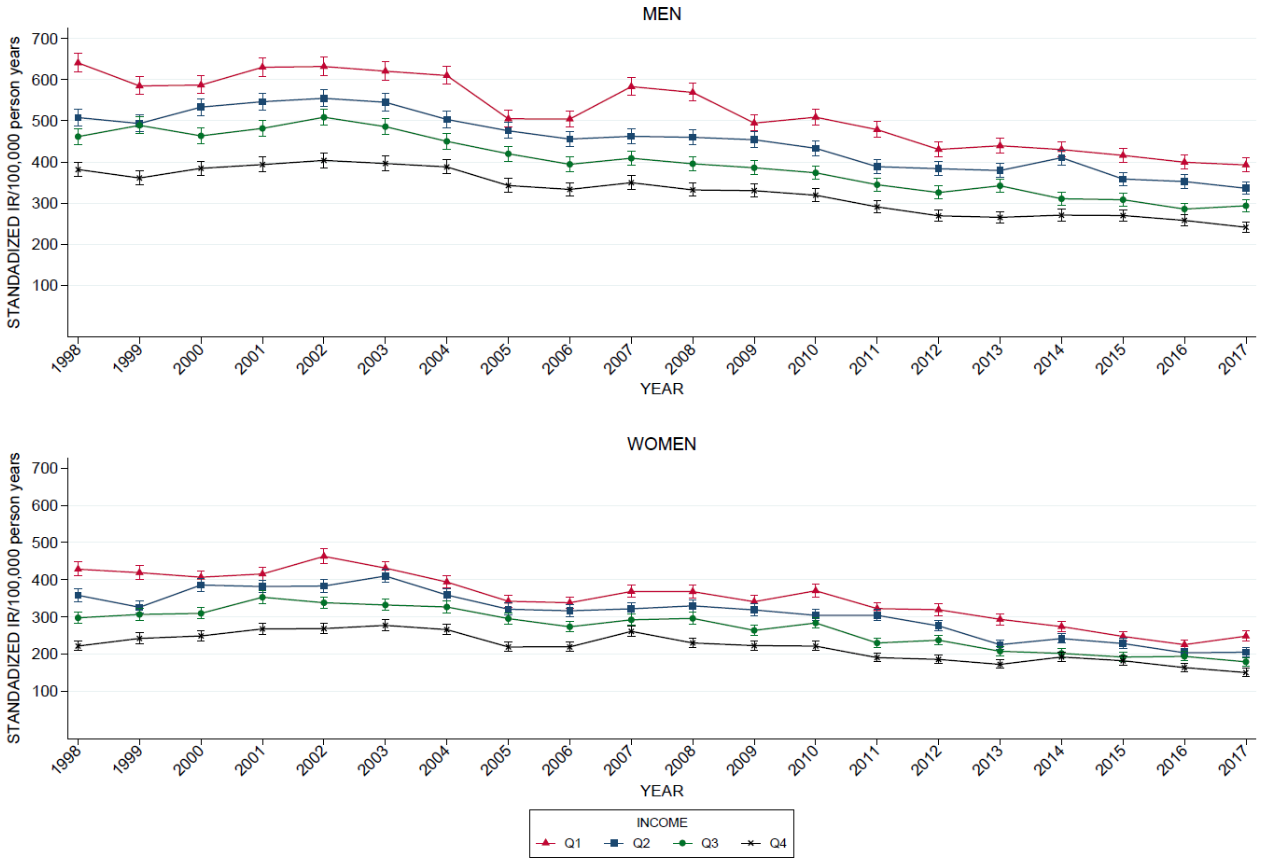

Table S5.1: Interaction analysis for time trends in incidence rates of ACS and dead possibly related to ACS according to personal income quartile.

	MEN	WOMEN
**Income**	**Coefficient [CI]**	**Coefficient [CI]**
Q1	-4.95 [-7.71; -2.19]	-5.35 [-8.12; -2.58]
Q2	-2.43 [-5.18; -0.32]	-4.14 [-6.90; -1.38]
Q3	-3.19 [-5.93; -0.46]	-2.90 [-5.65; -0.16]
Q4	*ref*	*ref*

The results are stratified by sex. Abbreviations: ACS: Acute Coronary Syndrome, CI: 95% Confidence interval, Q: quartile,

Figure S5.2: Standardised Incidence rate ratio of ACS and dead possibly related to ACS according to personal income quartile. *The results are stratified by sex. Abbreviations: ACS: Acute Coronary Syndrome, Q: Income Quartile from low (1) to high (4), which is the reference group, based on the income the year before the diagnosis, Ref: reference group. Analyses were standardised according to year, sex and age-group.*

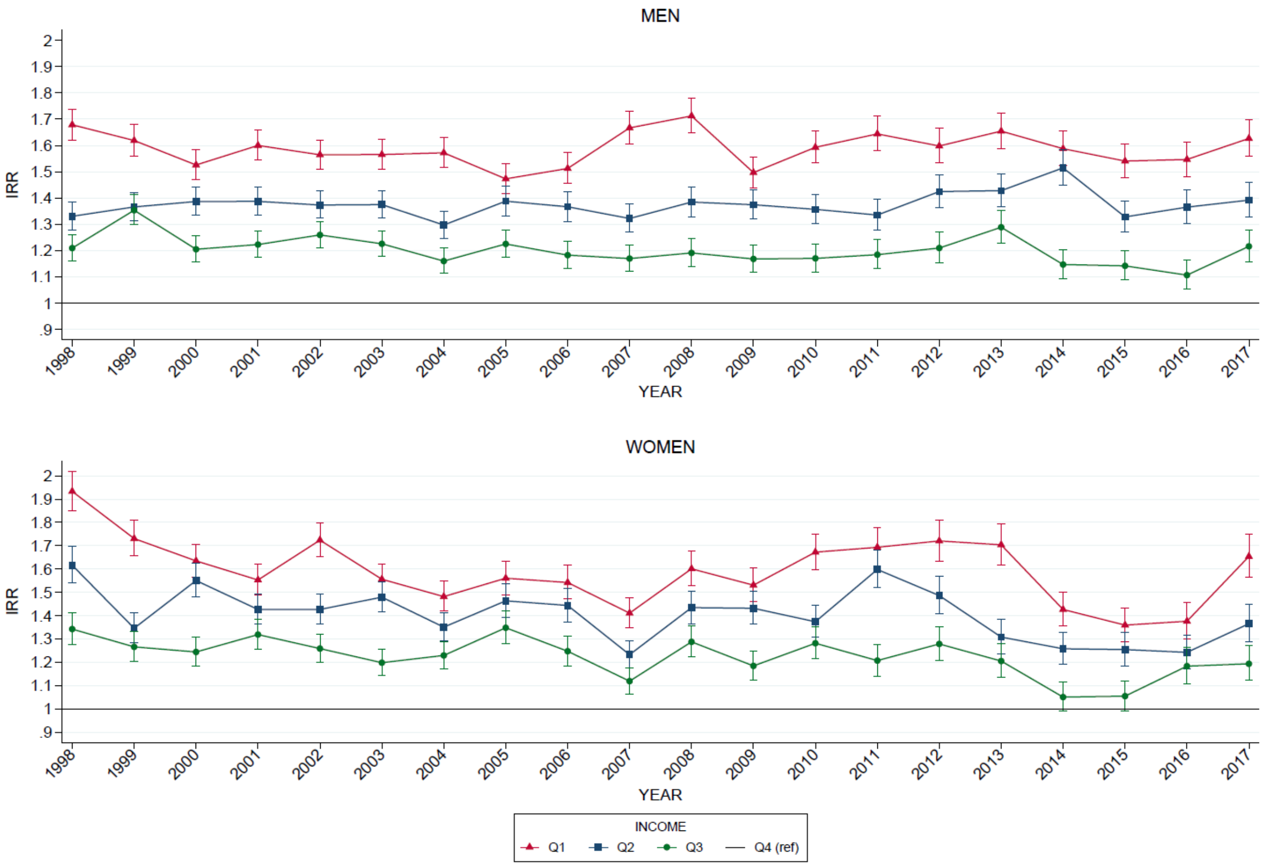

Supplement: ckad139_Supplementary_Data [file ckad139_supplementary_data.pdf]
